# What Kind of Dressing Is Important to Ensure Wound Healing With the Application of Platelet-Rich Plasma in Chronic Ulcers?

**DOI:** 10.7759/cureus.56758

**Published:** 2024-03-23

**Authors:** Ashraful Hoque

**Affiliations:** 1 Blood Transfusion, Sheikh Hasina National Institute of Burn and Plastic Surgery, Dhaka, BGD

**Keywords:** platelet-rich plasma, chronic wound management, platelet-rich fibrin (prf), platelet-rich plasma (prp), saltwater dressing, growth factors, povidone iodine

## Abstract

Wound healing is an intricate process of tissue regeneration that depends on the simultaneous presence of immunological and microenvironmental factors. The significant role of platelets and their granules in the wound-healing process has led to extensive research on their potential as a therapeutic intervention in different areas, including chronic wounds and aesthetic therapies. Saltwater aids in purification and promotes healing by utilizing osmosis. Sodium chloride, the chemical component present in salt, induces the extrusion of fluids from cells upon contact. If the liquids in issue are bacterial, they will also be ejected, assisting in the cleansing of the skin. Desiccation, often known as the drying out of injured cells, is well-known for its antibacterial properties and subsequent ability to reduce inflammation. This case series aims to investigate the advantages of using saltwater dressing following platelet-rich plasma therapy for chronic wounds.

## Introduction

The skin, which is the largest organ of the human body, has been shaped throughout thousands of years of development [1]. It accounts for 15% of the total body mass. The skin is a versatile and resilient organ that acts as a barrier, shielding internal organs and safeguarding the body against physical harm, microbial diseases, and environmental extremes. Furthermore, any disparity in the skin's structural, anatomical, and functional integrity might lead to the development of wounds. Wound healing is a complex biological process that restores the normal structure and function of injured tissue [2]. When tissue is injured, a diverse range of cells, growth factors, cytokines, and chemokines beneath the layers of skin work together to initiate and carry out several stages of the wound-healing process. These stages include hemostasis, inflammation, angiogenesis, epithelization, and tissue remodeling [3].

Platelets have traditionally been believed to serve hemostatic functions for the past 40 years [4]. A recent study has revealed that platelets have significant involvement in tissue regeneration and immunological processes [5]. The fundamental basis for utilizing platelet-rich plasma (PRP) in managing persistent wounds lies in the molecular constitution of thrombocytes, which possess a high concentration of growth factors and other physiologically active components. PRP acts as a scaffold for cells, hence enhancing the wound-healing process [6]. The process of harvesting PRP is quite simple, involves minimum invasion, and has the potential to be accessible to every patient.

The act of cleaning wounds or managing them with antiseptics has a history characterized by two contrasting aspects, rooted in tradition and scientific knowledge. It is a crucial component of managing both acute traumatic wounds and chronic wounds. Currently, there are no diagnostic tools available for healthcare practitioners to determine the bacterial load in a wound that has the potential to cause an infection. Therefore, it is widely considered that all wounds should be cleansed to reduce the number of germs present to a level that can be effectively controlled by the body's natural defenses. The selection of a purifying agent, meanwhile, continues to be a subject of dispute. The effectiveness of antiseptics has been closely examined [7-9].

The use of dressing materials as preventive anti-infective agents for open wounds, such as cuts, scrapes, burns, and long-lasting sores, has been a subject of heated debate for many years. Referring to cytotoxicity evidence, numerous publications have cautioned against their application on open wounds.

The significance of PRP to current medical science is unparalleled, particularly in the domain of wound healing. However, even after the application, it is necessary to regularly dress the wound to minimize the risk of infection and optimize its effectiveness. Here, we shall examine several instances in which PRP was utilized and the effectiveness of saltwater dressing was verified. By conducting a thorough follow-up and analysis of the data, I will demonstrate the significant advantages it offers compared to other traditional dressing approaches.

## Case presentation

Method

The study comprised patients who had been suffering from diabetic foot ulcers for a minimum duration of three months. Each of them received standard treatment, specifically normal dressing, and none of them has undergone any prior PRP therapy, vacuum-assisted closure (VAC) therapy, or hyperbaric oxygen therapy (HBOT) treatment. Before treatment, they had diabetes screening (glycosylated hemoglobin, HbA1c). Out of them, two were determined to be governed and one was determined to be unregulated.

A complete blood count (CBC) was conducted before PRP production, and thrombocytopenia was ruled out in all cases. An anxiolytic (tablet clonazepam 0.5 mg) was administered to them to ensure a restful night's sleep before their scheduled procedure. It is recommended to refrain from consuming a large meal within two hours before blood collection [10]. This is because the presence of chylomicrons in the blood can enhance the release of growth factors from platelets.

Acid citrate dextrose (ACD; Terumo Penpol Private Limited, Thiruvananthapuram, India) was taken in a ratio of 1:9 in a 20-cc syringe. Blood was collected from the antecubital vein with a 19-g scalp vein needle. During blood collection, gentle pressure was applied to the plunger of the syringe so as not to damage the blood platelets.

PRP was prepared by taking the blood collected from the syringe in a 15-ml sterile falcon tube and using a Thermo Fisher Scientific Sorvall ST16 centrifuge machine (Waltham, MA). Centrifugation at 2500 RPM for six minutes first separates the red cells and plasma. Another sterile falcon tube receives it via micropipette. The final product was again centrifuged at 3400 RPM for three minutes. The platelet count showed a three- to four-fold rise from the original value and reached around 700,000 to 800,000 units. The initial platelet count of the first patient was 230,000, the second patient had a count of 275,000, and the last patient had a count of 305,000 units.

Case 1

**Figure 1:**
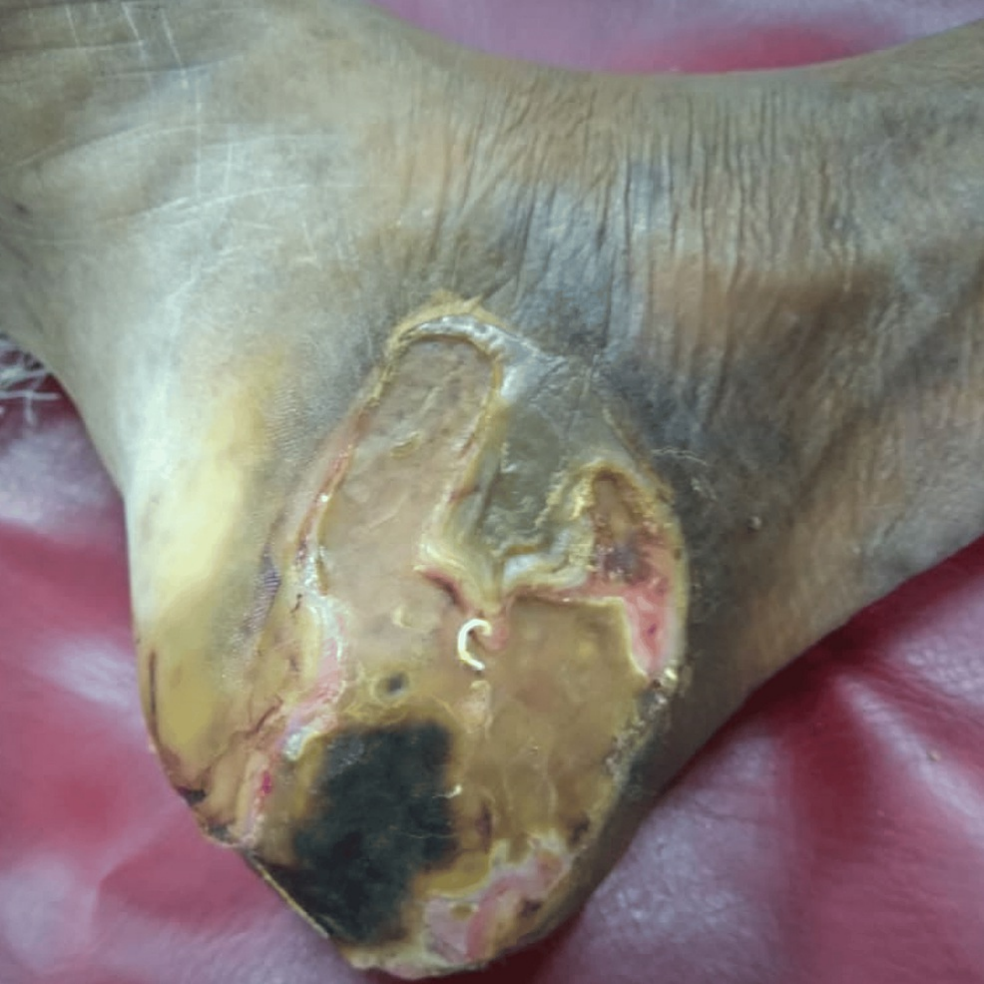
Gangrene ankle The wound was healthy and no granulation tissue was seen. There was no sinus tract and the undermined area was not present.

A 65-year-old male patient was hospitalized due to a left ankle infection (Figure 1). Due to the development of gangrene, it was necessary to amputate the affected area. As a result of persistent and unregulated diabetes, the amputation was not healing anymore. Regular application of povidone-iodine was done three times a week for dressing purposes.

**Figure 2:**
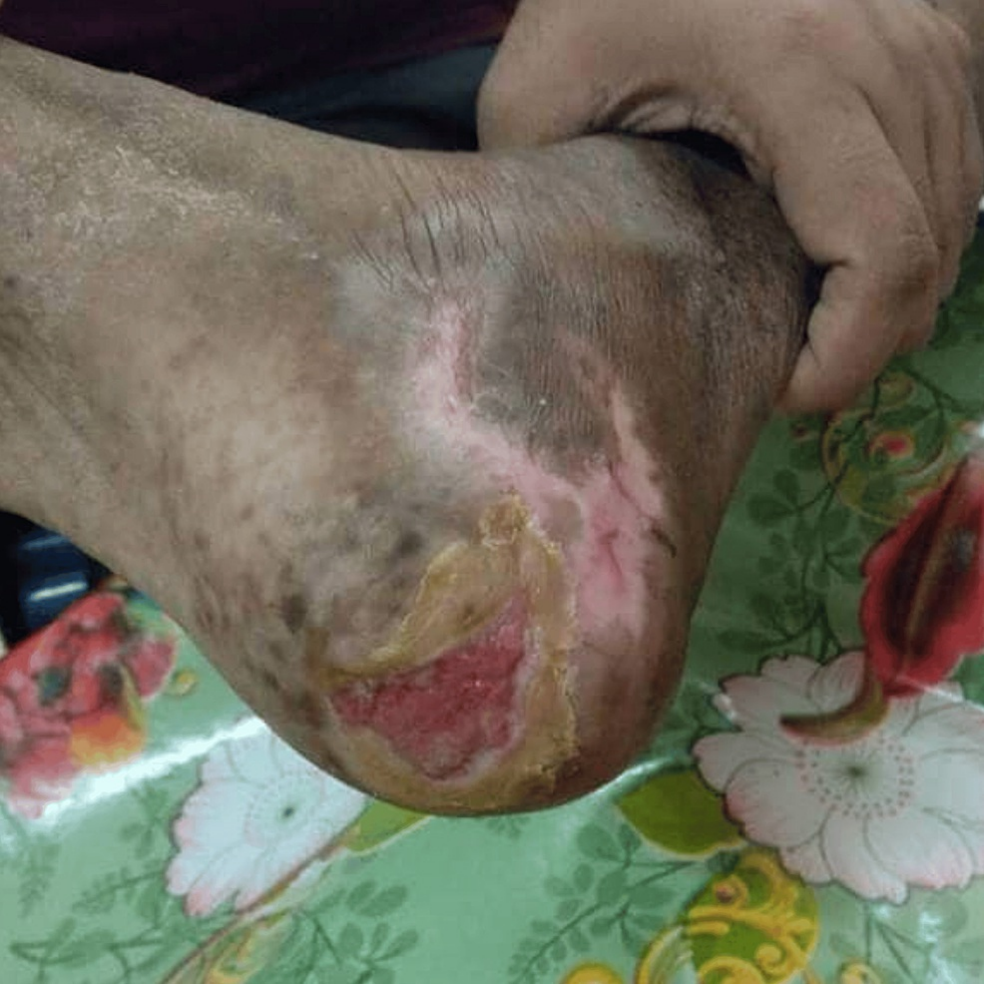
Healed ankle The wound size was decreased dramatically.

PRP was administered to this patient, flushed with normal saline. About 12 ml of PRP was given with a 3-cc syringe around the wound site. Then, every day, the feet were immersed in warm water with salt mixed for 15-20 minutes; especially, the wound was properly immersed in the water. Then the feet were dried with a clean cloth. This was done once a day, every day. No other materials were used. Since the patient was hospitalized, the gauze piece was covered. Within 21 days, the patient's wound was almost back to normal (Figure 2).

Case 2

**Figure 3:**
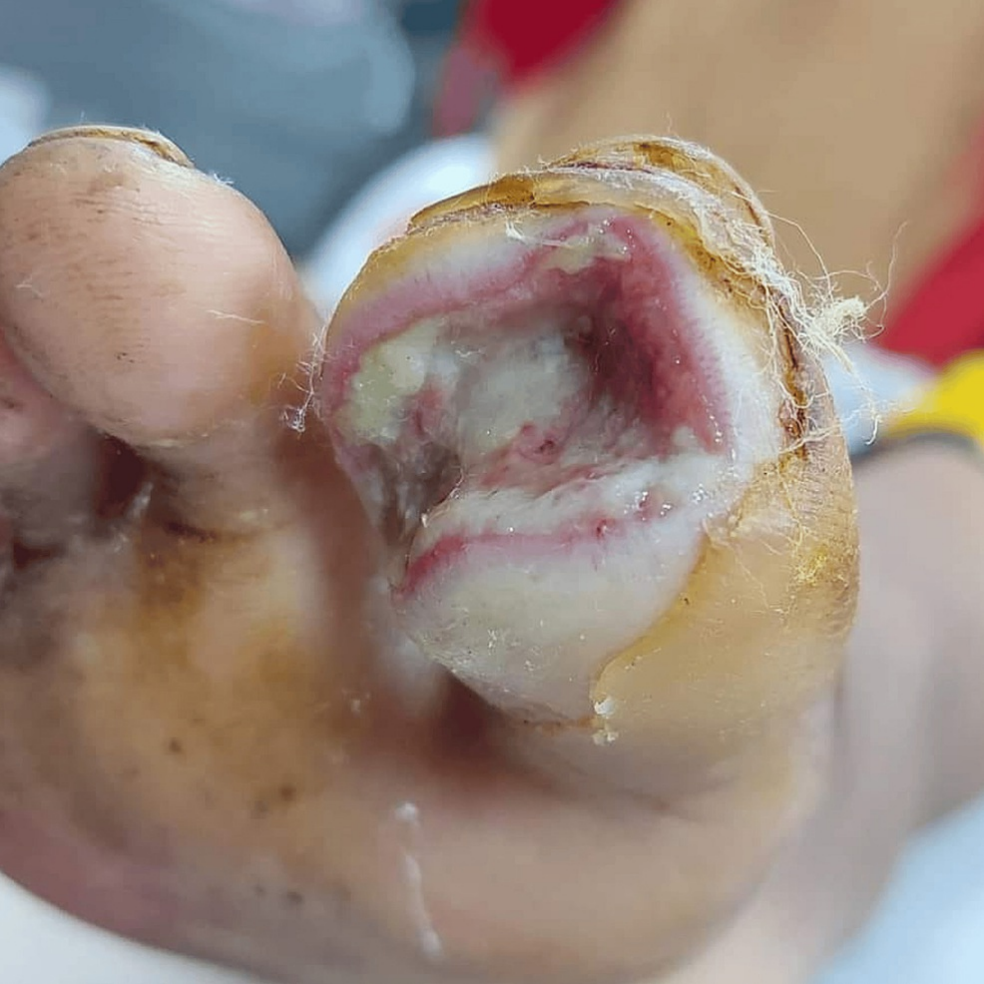
Infected great toe The wound was unhealthy and small granulation tissue was seen around the margin. No sinus tract or undermined area was present.

A female patient, aged 37, came in with an infection in her right big toe (Figure 3). Conventional dressing was not yielding the desired outcomes. Following three months, during which the toe developed an infection, the doctor recommended that she undergo PRP treatment. Upon assessment, it was determined that her diabetes was well-managed with oral medicine, with a HbA1c level below 7.

**Figure 4:**
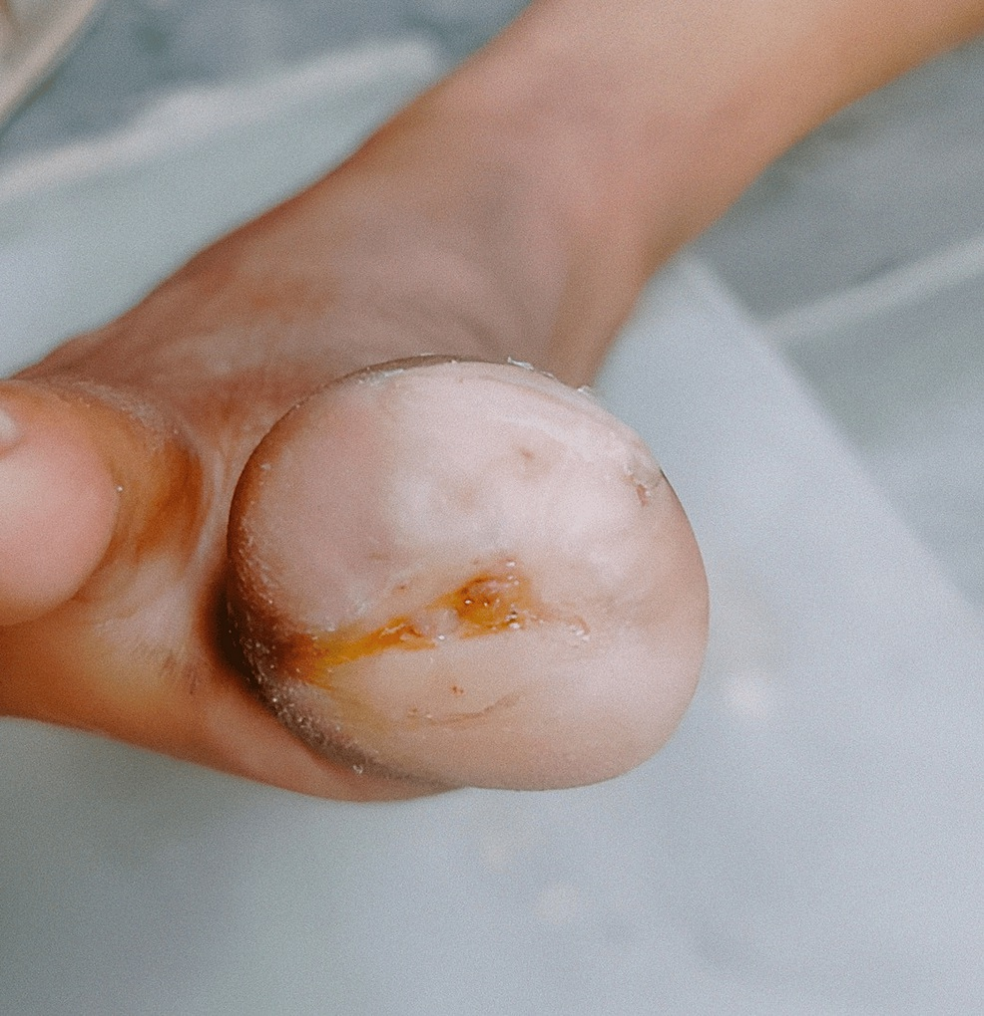
Healed great toe

As in the previous case, the wound was cleansed with normal saline, and PRP (8 ml) was applied to the aspiration side of the wound. In the same way, every day, the wound was cleaned with warm water and salt for 15-20 minutes and dried with a clean cloth. Within 18 days, the patient's wound was almost back to normal (Figure 4).

Case 3

**Figure 5:**
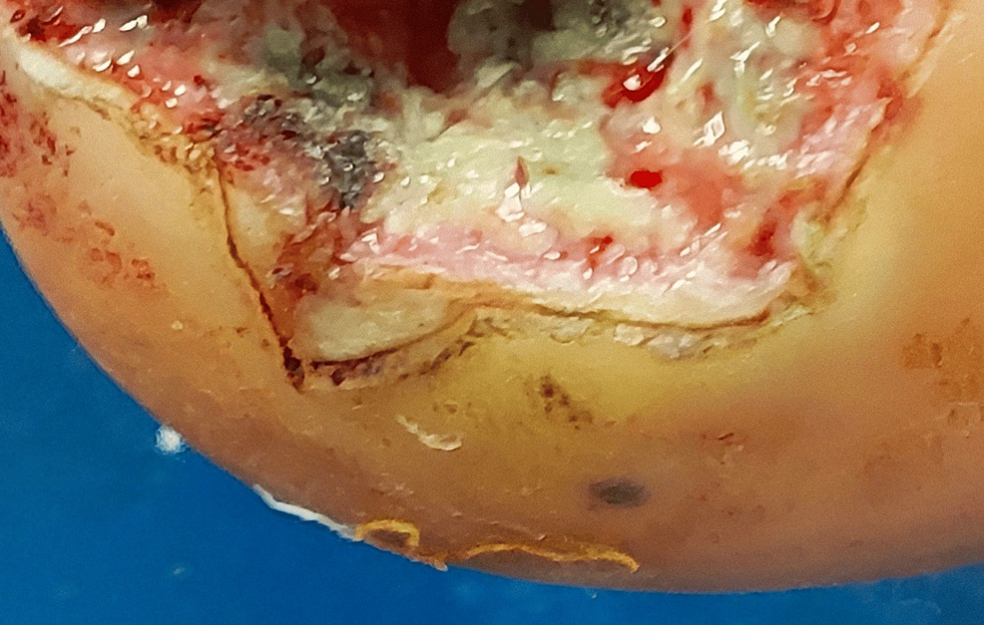
Infected sole The wound area was unhealthy, full of pus with few bleeding spots. No sinus tract or undermined area was present.

Patient number three had an injury to their right ankle when a fragment of iron pierced through it while walking. Upon removal, the region ceased to be arid. Despite consistent dressing, it was observed that the dimensions of his wound were expanding. Therefore, numerous components need to be surgically excised. However, even this failed to equate to the level of liberty. The circumstances were such that it was advised to amputate the ankle (Figure 5).

**Figure 6:**
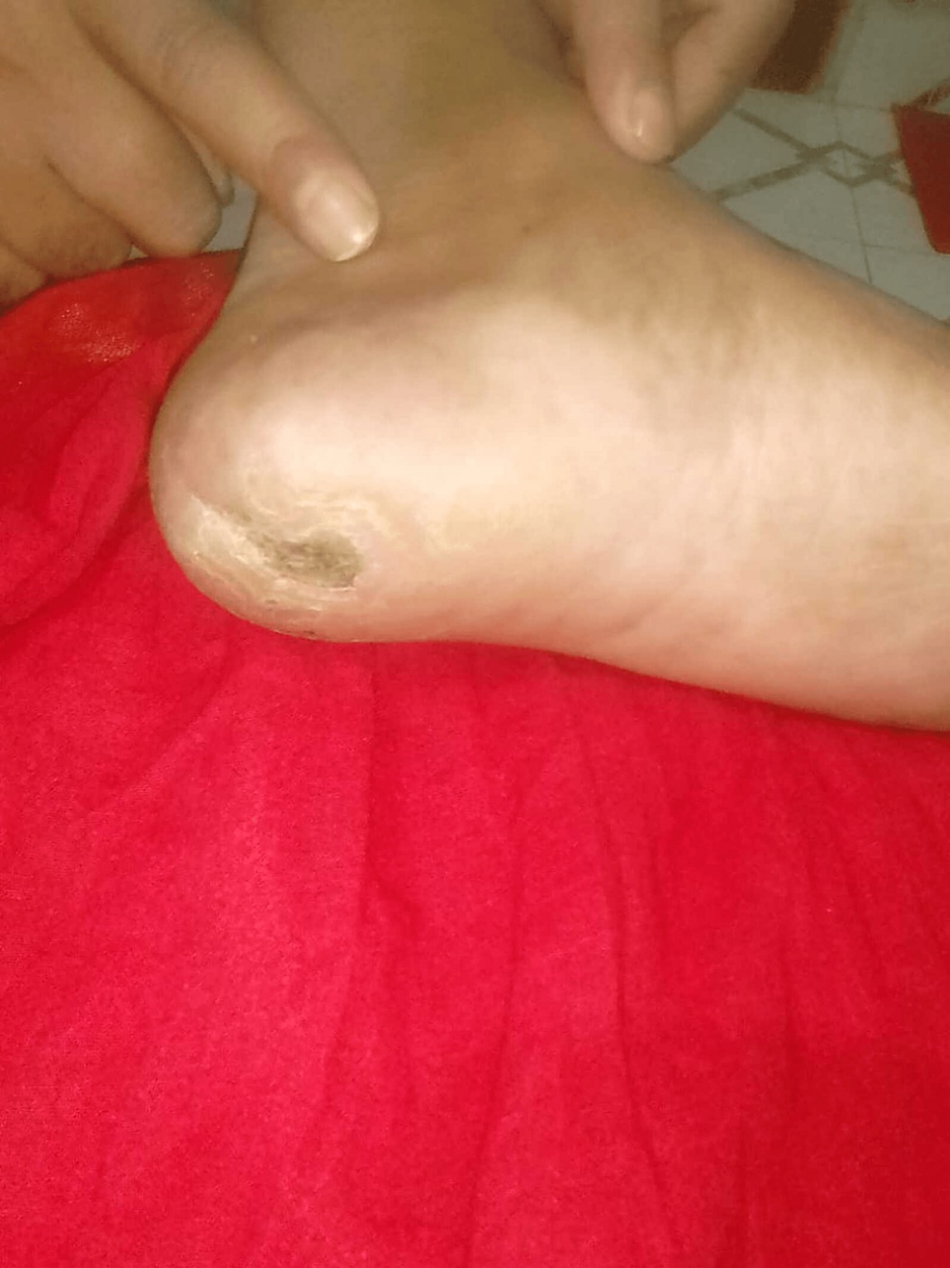
Healed sole

The patient's diabetes remained well-managed during his hospitalization. PRP was administered following the prescribed procedure. Subsequently, ordinary curd was immersed in boiling water along with salt. The patient's wound typically underwent healing within approximately 28 days. Nevertheless, due to the extensive removal during the prior procedure, the subsequent healing process results in a greater presence of fibrous tissue. No intervention was necessary, as it did not impede the patient's regular movements (Figure 6).

## Discussion

The presence of chronic wounds is a characteristic of a disrupted wound-healing process. These wounds are mainly observed in people who have diabetes, vascular diseases, hemoglobinopathies, neuropathies, and older people. This condition of wound mismanagement is typically characterized by repeated occurrences and, in some cases, may necessitate drastic measures, such as limb amputation. In severe instances, it can even result in death. Metabolic illnesses, such as obesity or type 2 diabetes, are characterized by the presence of inflammatory cells in the granular tissue layer, which have a significant impact on the immune system and can alter the process of wound healing [11].

Platelet-derived products have been effectively utilized in many sub-fields of regenerative medicine since 1990. In recent years, PRP-based treatments have gained significant attention for their potential to address several clinical issues, including wound healing, skin and bone regeneration, ophthalmology, ulcer treatment, burn healing, muscle restoration, and more [1].

PRP therapies help wounds heal faster by sending a lot of growth factors, cytokines, and chemokines to the wound site. These are important for tissue regeneration. These factors also control inflammation, angiogenesis, the production of extracellular matrix, and the remodeling of newly produced tissue. Elevated levels of growth factors also enhance the regeneration of epithelial and endothelial cells and the production of collagen. The PRP-based treatment method was employed for the initial time to address chronic leg ulcers, leading to the successful creation of vascularized connective tissue. In addition to having the potential to be antimitotic, wound cleaners have the potential to have an unfavorable effect on the repair of normal tissue [12].

PRP specifically interacts with various cells and tissue contexts, hence regulating cellular signaling and potentially exerting a paracrine influence [13]. In the case studies I provided, PRP was administered to the wound site. Growth factors exert their effects at the location of the wound through a paracrine mechanism, leading to the observed outcome.

PRP with a platelet count of at least 1,000,000 platelets per microliter in a 5-milliliter plasma sample has been associated with enhanced healing [14]. The outcomes of these case studies are similar.

In this study, the wound was dressed with a warm saline solution because earlier traditional dressing did not get results. Additionally, many studies have shown that chemical dressings often disrupt the normal growth of healthy tissue. Iodine products significantly decrease the number of bacteria and inhibit their growth through bactericidal and bacteriostatic mechanisms. They are active against most kinds of bacteria, particularly those commonly found in chronic wound care [15]. Although iodine products have antibacterial benefits, their clinical application for wound treatment has shown various possible drawbacks and yielded conflicting outcomes [16]. For wound cleansing solutions to be efficient, they must not be poisonous to human tissues, they must continue to be effective even when organic material is present, they must lower the number of germs, they must not produce sensitive reactions, and they must be widely available and cost-effective. It is recommended that we use sterile water, normal saline water, or even just plain old tap water as cleansing agents [17].

In each case, success was achieved with just one application of PRP. No second application was required, and the desired improvement was seen within a month. Many studies have applied more than once. Additionally, follow-up over months is required in those areas.

A 57-year-old patient had PRP treatment at weekly intervals, resulting in complete healing after 28 days [18]. In my study, only one-time PRP use was enough for healing. In a different trial, adipose tissue was combined with PRP in a male patient who was 65 years old. It attained success after 15 weeks of follow-up [19], but in my study, healing occurred within 28 days. Another study showed that the significance of education in exerting a favorable influence on patient behavior was considerable [20]. A disparity was identified between the level of knowledge and practice among patients. In my study, I found similar results. Education of the patients influences a lot the outcome of any biological application.

The success or failure of any biological therapy requires a large sample size. No strong comment can be made with only three samples. This is the weakest aspect of this study. However, the patient can use the study's dressing method at home. As a result, I believe many things will become easier, including financial savings for the patient. However, research with larger samples and RCTs is needed to establish this.

## Conclusions

Chronic wounds are a big challenge for our medical science, especially due to diabetes, because if wounds cannot be treated properly, amputation is necessary most of the time. It not only cripples the patient but also largely cripples their family. There is a lot of work going on with the use of PRP and a lot of success. However, no final guidelines have been published yet for its preparation, which is a major negative point. In addition, it is not only necessary to apply it but also to do regular dressings. Diabetic ulcer patients spend so much money on their treatment that sometimes it becomes a big challenge to manage the treatment. The dressing procedures discussed in this case series are simple for any patient. Studies with such small numbers of patients can never provide definitive conclusions. This requires larger studies.
